# Rational Emotive Behavior Therapy (REBT), Irrational and Rational Beliefs, and the Mental Health of Athletes

**DOI:** 10.3389/fpsyg.2016.01423

**Published:** 2016-09-20

**Authors:** Martin J. Turner

**Affiliations:** Centre for Sport, Health and Exercise Research, Staffordshire UniversityStoke-on-Trent, UK

**Keywords:** irrational beliefs, rational beliefs, mental health, performance, sport psychology, REBT, emotions, behaviors

## Abstract

In this article Rational Emotive Behavior Therapy (REBT) is proposed as a potentially important framework for the understanding and promotion of mental health in athletes. Cognitive-behavioral approaches predominate in the provision of sport psychology, and often form the backbone of psychological skills training for performance enhancement and maintenance. But far from being solely performance-focused, the cognitive-behavioral approach to sport psychology can restore, promote, and maintain mental health. This review article presents REBT (Ellis, 1957), the original cognitive behavioral therapy, as a valuable approach to addressing mental health issues in sport. REBT holds that it is not events that directly cause emotions and behaviors. Rather, it is one’s beliefs about the events that lead to emotional and behavioral reactivity. Further, REBT distinguishes between rational and irrational beliefs, and suggests that in response to failure, maltreatment, and misfortune, people can react with either healthy or unhealthy emotional and behavioral responses. The extant research indicates that irrational beliefs lead to unhealthy negative emotions, a range of pathological conditions, and a host of maladaptive behaviors that undermine mental health. Therefore, REBT proposes a process for the reduction of irrational beliefs and the promotion of rational beliefs. The use of REBT in sport is seldom reported in literature, but research is growing. This review article proposes three important areas of investigation that will aid the understanding of irrational beliefs and the application of REBT within sport. These areas are: (1) the influence of irrational beliefs and REBT on the mental health of athletes, (2) the influence of irrational beliefs and REBT on athletic performance, (3) the origins and development of irrational beliefs in athletes. Each area is discussed in turn, offering a critical and progressive review of the literature as well as highlighting research deficits, and recommendations to address each of the three areas of investigation.

## Introduction

The provision of sport psychology within sporting organizations and with athletes can be approached in many ways. At present, cognitive-behavioral approaches predominate, where imagery, self-talk, relaxation, concentration, and goal setting (known as ‘The Canon’) have been shown to be particularly effective in helping athletes to enhance and maintain athletic performance (Andersen, 2009). But far from being solely performance-focused, the cognitive-behavioral approach to sport psychology can restore, promote, and maintain mental health. Indeed, many consider sport psychology to be much more than the provision of psychological skills training (PST), recognizing the role sport psychology could play in the mental health of athletes. Also, many recognize the importance of viewing athletes as humans first, and athletes second, thus reinforcing a humanistic approach to helping athletes with self-defeating emotions and behaviors, inside and outside of their sport. This is not to say that sport psychologists should ‘treat’ athletes for mental illness; this is ethically beyond many practitioners’ professional competencies and occupational remit. However, providing that the practitioner is trained and competent in the use of counseling approaches, it is possible to work with athletes on deeply held attitudes and beliefs that positively influence not only sports performance, but also mental health.

One humanistic cognitive-behavioral approach that is receiving growing attention in sport literature is Rational Emotive Behavior Therapy (REBT; Ellis, 1957). REBT is considered to be the original cognitive-behavior therapy (CBT) by many scholars, and was developed by Dr. Albert Ellis in the 1950s and was driven in part by Ellis’ desire to conceive of a more effective psychotherapy that addressed some of the shortcomings of psychoanalysis (Froggatt, 2005). Inspired primarily by the Stoic philosophers, REBT holds that it is not events that directly cause emotions and behaviors. Rather, it is one’s beliefs about the events that lead to emotional and behavioral reactivity. This is a common cognitive-behavioral philosophy shared across various approaches. REBT places this central idea or philosophy into an ABC framework where the event is represented by the letter A (activating event or adversity), the beliefs are allocated the letter B, and finally emotions and behaviors are represented by C (consequences). Not only does this ABC framework hold up scientifically when considering the role of cognitive appraisal in the generation of emotion (David et al., 2002), it also facilitates therapy, as it is a non-complex and memorable way for clients to understand the antecedents to their emotions and behaviors. Most prominently, it enables clients to realize that it is not outside events (A) that cause their dysfunctional reactions (C), it is their irrational beliefs (B), and thus, they are in control of how they respond to adversity because they can have autonomy over their beliefs.

Theorists and practitioners (e.g., Ellis, 1994; David and McMahon, 2001) assert that rational and irrational beliefs are types of ‘hot’ cognition (Abelson and Rosenberg, 1958) or evaluative cognition (David et al., 2005b). Cold cognitions describe how an individual develops representations of situations, whereas hot cognitions refer to the evaluation of cold cognitions, or appraisals (David and McMahon, 2001; David et al., 2002). Rational and irrational beliefs are also considered to be ‘deep’ cognitions akin to schemas or core beliefs, which are difficult to consciously access. Rational and irrational beliefs, if they are indeed schemas, are complex structures that represent a person’s constructed concepts of reality, and behavioral responses to that reality (David et al., 2005b). Therefore, emotions emerge as a result of cold cognitions that deem a situation to be motivationally relevant and motivationally incongruent, mediated by rational and irrational beliefs (hot cognitions). Put another way, the ability for A (activating event; cold cognition) to cause C (emotional and behavioral response) is dependent on B (rational and irrational beliefs; hot cognition). Hence, the ABC philosophy that informs REBTs theoretical and therapeutic approach serves to guide treatment and capture the mechanisms driving emotional responding.

Rational Emotive Behavior Therapy distinguishes itself from other cognitive-behavioral approaches by placing irrational and rational beliefs at its core. In REBT rational beliefs are defined as beliefs that are flexible, non-extreme, and logical (i.e., consistent with reality), and in contrast, irrational beliefs are rigid, extreme, and illogical (i.e., inconsistent with reality). Specifically, there are four types of rational and irrational beliefs. Rational beliefs comprise a primary belief (preferences) and three secondary beliefs (anti-awfulizing, high frustration tolerance; HFT, and self/other acceptance). In sport, preference beliefs could reflect a belief that “I want to be successful but that does not mean I have to be.” An anti-awfulizing belief could be that “if I do not succeed it would be bad, but not awful.” An example of HFT is “failure is difficult, but not unbearable.” A self-acceptance belief could be “when I fail, it is bad, but does not mean that I am a complete failure,” whereas other-acceptance could reflect a belief that “when coaches treat me poorly it is bad, but does not prove they are bad people.” Irrational beliefs comprise a primary belief (demandingness) and three secondary beliefs (awfulizing, low frustration tolerance; LFT, and self/other depreciation). In sport, demandingness beliefs could reflect a belief that “I want to be successful and therefore I must.” An awfulizing belief could be that “if I do not succeed it will be awful” An example of LFT is “it is unbearable to fail.” A self-depreciation belief could be “when I fail, it means that I am a complete failure,” whereas other-depreciation could reflect a belief that “when coaches treat me poorly, it proves they are bad people.”

In REBT a binary model of distress is proposed whereby healthy negative emotions (HNEs) associated with adaptive behaviors stem from rational beliefs, whilst unhealthy negative emotions (UNEs) associated with maladaptive behaviors stem from irrational beliefs. UNEs are associated with very unpleasant physical symptoms (chronic and severe) and usually motivate behaviors that work against goal attainment. In contrast, HNEs facilitate goal accomplishment as they are associated with some unpleasant physical symptoms (acute and mild) and motivate behaviors that facilitate goal attainment. HNEs and UNEs are not necessarily distinguished by the intensity of the emotion, rather, they are qualitatively different. In other words, it is not that unhealthy anxiety is less intense than healthy anxiety, or that they are just two versions of the same emotions. It is more accurate to consider them to be different emotions altogether as they drive different behaviors (or action tendencies). This binary model of distress (David et al., 2005a) is in line with research indicating that negative emotions are not always dysfunctional, and can be adaptive (e.g., Kashdan and Biswas-Diener, 2014).

Unsurprisingly, the central aim of REBT is to reduce irrational beliefs in favor of rational beliefs, encouraging decreases in UNEs and increased in HNEs (Ellis and Dryden, 1997). This is done using a systematic disputation (D) process, which entails the practitioner helping the client to challenge specific irrational beliefs (Dryden, 2009). The client is asked to consider whether there is any evidence for their belief, whether it is logical or consistent with reality, and whether the belief is pragmatic or helpful. Once the irrational belief has been disputed, a rational alternate belief is constructed, in line with theory and in collaboration between client and practitioner, a step labeled E (effective new belief). Depending on the motivation of the client, REBT can be completed briefly in as little as five sessions for clearly defined issues but more long-term REBT is recommended for more complex issues (Digiuseppe et al., 2014). However, longer REBT (in terms of minutes) is considered more effective, having greater impact on treatment outcomes (Lyons and Woods, 1991; Gonzalez et al., 2004). The precise processes of using REBT with athletes can be found in the extant literature (Ellis and Dryden, 1997; Turner and Barker, 2014), but the efficacy of the ABCDE process, and more broadly REBT, has been supported in hundreds of research articles (David et al., 2005b) in both clinical and non-clinical populations, youths and adults (David and Avellino, 2002), and by three meta-analyses (Lyons and Woods, 1991; Engels et al., 1993; Gonzalez et al., 2004). Practitioners wishing to ethically adopt REBT within their practice should acquire professional competencies by completing a recognized and official REBT course, and also maintain their knowledge and skills via peer support groups. Because there is a paucity of research reporting the use of REBT with athletes, meta-analyses conducted with non-athletes provides acceptable, but not strong, justification for the use of REBT with athlete populations. However, sport literature has started to report the use of REBT in athlete populations.

## REBT and Irrational Beliefs in Sport

One of the advantages of practicing and studying sport psychology is the exposure to a broad range of psychological approaches, many of which that have their groundings in cognitive behavioral approaches. As already mentioned, ‘The Canon’ (Andersen, 2009) comprises cognitive-behavioral techniques that are effective in helping athletes approach and manage training and performance situations by helping them control cognitions, emotions, and behaviors. As an REBT practitioner it is possible to use The Canon and REBT alongside one another, with REBT also advocating techniques such as imagery within its framework. However, the author finds that REBT is particularly useful for accessing, challenging, and changing more deeply held beliefs and philosophies than the techniques included within The Canon. For example, following REBT, athletes with rational beliefs still get anxious (healthy anxiety) about competing and The Canon provides useful strategies for reducing symptoms such as rumination and debilitative arousal. But some athletes require deeper-level work in order to counteract core irrational beliefs that drive unhealthy emotions and behaviors that may be more effectively treated through REBT.

It is also important to recognize that REBT advocates a humanistic philosophy that within sport, the author operationalizes as ‘human first, athlete second.’ REBT is humanistic and thus focuses on the person, not just the athlete, and also not just the performance. Therefore REBT is applicable for a vast range of athlete issues apart from performance issues, such as career transition, personal life issues, and eating disorders. The goal of REBT is to enhance and maintain emotional and behavioral functionality, which then helps to drive long-term goal achievement. In the context of sport, where the result is often the most important factor and a quick fix is tempting, athlete mental health is sometimes forgotten. It is important to recognize that REBT is also a preventative approach that can bolster rational beliefs and mental health, and is not just about providing a solution to irrational beliefs and mental ill-health. REBT advocates a change in the athlete’s philosophy of life, so that the individual can face many sport and life situations with rational beliefs and associated functional cognitions, emotions, and behaviors. This also helps athletes to self-manage emotions once they have been suitably trained to use REBT independently and competently. Sport, and many other performance contexts, can be too reactive to problems, which can cause sport psychology provision to be seen as remedial, rather than a core part of athlete support. Nonetheless, the growth of sport psychology has helped practitioners integrate well-established and also novel approaches into their practice, which in the case of REBT is reflected in the recent attention it has received in sport psychology literature.

Rational Emotive Behavior Therapy can be delivered in time-constricted and access-restricted situations, typical of some sporting environments. Therefore the modes of delivery typical in REBT such as group therapy, education, and one-to-one counseling, fit well within the provision of sport psychology. Perhaps many sport psychology practitioners use REBT within their practice, but current literature has sparse examples of REBT being used with athletes. The writing concerning the use of REBT in sport has been focused on case-study reflections (e.g., Marlow, 2009) and single-case designs (e.g., Turner and Barker, 2013). For example, Bernard (1985) provides a very detailed description of his work applying a rational-emotive training program with Australian Rules Football players. Delivered in a group setting, the program included REBT education and also broader themes such as concentration training and goal setting. Bernard (1985) reports that the athletes were better able to control their thoughts to directly influence performance. However, no performance markers were attained and no control-group was present as this work was not a research study, deeming the extent to which the program influenced actual performance impossible to ascertain. A similar approach was taken by Marlow (2009) who applied REBT with a youth ten-pin bowler, again within a broad program of psychological skills, reporting positive performance effects alongside adaptive behavioral changes.

Away from reflective case-study approaches, there have been a handful of studies that focus on changes in relevant dependent variables through the application of REBT. Elko and Ostrow (1991) applied REBT with six gymnasts and found reduced anxiety in five and enhanced performance in three of the participants. The lack of performance gain in three of the gymnasts is feasibly attributed to circumstantial events, but may indicate that the promotion of rational beliefs does not necessarily improve athletic performance. In another study, five lecture-based REBT sessions were provided to youth soft-tennis players, with results indicating that cognitive-anxiety was significantly reduced (Yamauchi and Murakoshi, 2001). However, this study is written in Japanese, has not been translated, and therefore the author has been unable to discern the precise details of the study. One study examined the efficacy of REBT for managing trait and state anxiety direction, and ten-pin bowling performance, compared to an imagery and relaxation intervention, and a placebo intervention (Larner et al., 2007). As is typical in REBT, the intervention focused on changing participants’ interpretations of competition circumstances, cognitions, behaviors, and feelings by disputation of underlying beliefs. The relaxation and imagery intervention comprised rehearsal of alternate physiological and mental states during competition, and the placebo intervention emphasized general attention and reflective counseling. The REBT intervention reduced irrational thinking significantly more than the comparison interventions, which is to be expected. However, REBT also significantly moderated negative directional interpretations of trait and state anxiety symptoms, and improved performance to a greater extent than the comparison interventions. Another study focused on the specific irrational belief of LFT (Si and Lee, 2008) and applied REBT and mental skills training with an Olympic table tennis athlete. Using coach and teammate evaluation and video analysis, results showed a reduction in behaviors related to LFT, and performance enhancement in competitions.

More recent research has emerged that has adopted single-case designs to assess the effectiveness of REBT with athletes. In a study by Turner and Barker (2013), four elite youth cricketers received three one-to-one REBT counseling sessions regarding their performance anxiety. Results showed a significant reduction in irrational beliefs and cognitive anxiety when REBT was applied, but no objective performance markers were collected and therefore the impact of REBT on performance was not evidenced. Two further studies (Turner et al., 2014, 2015) showed that REBT education sessions were able to significantly reduce irrational beliefs in elite soccer athletes. However, when REBT education was applied in a single session, reductions in irrational beliefs were short-term, returning to baseline levels at a follow-up timepoint (Turner et al., 2014). Whereas REBT education applied in three sessions yielded longer-term reductions in irrational beliefs, lending support to the idea that REBT is not a quick fix. Again, although in both studies subjectively athletes felt that the REBT helped them improve emotional control and performance, no objective markers of performance were sought. More recently (Cunningham and Turner, 2016), REBT was used with three semi-professional Mixed Martial Arts athletes on a one-to-one basis, to reduce irrational beliefs, in particular self-depreciation, and increase unconditional self-acceptance. Results showed that two of the three athletes reported decreases in self-depreciation, and all three showed increases in unconditional self-acceptance (USA). Also, in a detailed case-study paper REBT was applied with a country-level archer across seven sessions (Wood et al., 2016), demonstrating decreases in irrational beliefs, and increases in rational beliefs, self-efficacy, perceived control, and objective competitive performance scores.

## Moving the Area Forward

As is evident in the short review of literature concerning REBT in sport, the research is scant, thus preventing the formulation of strong conclusions as to REBT’s efficacy within sport. Further, the research that exists has focused on the application of REBT with athletes in the field, and not on testing and validating the theoretical proponents of REBT in sport settings, or with athletes. The number of empirical research papers and practitioner reflections are growing in the sport and REBT literature, but most articles focus on how the application of REBT reduces irrational beliefs in athletes, with the use of social validation data to explore broader changes at an emotional and behavioral level. With the research in sport in its infancy, there are a number of areas in which future research should be directed. In this article the author presents three key areas in which further research should be invested in order to advance the understanding of irrational and rational beliefs and REBT in sport.

First, the influence of irrational and rational beliefs and REBT on the mental health of athletes should be more fully investigated. Although extant sport research has reported shifts in irrational and rational beliefs and emotional outcomes (e.g., anxiety; Turner and Barker, 2013), research is yet to examine the effects of irrational beliefs and rational beliefs on broader mental health outcomes in athletes. Second, given that sport is a performance-driven industry, the influence of irrational and rational beliefs and REBT on performance should be more fully empirically tested. While the extant research provides growing support for the applicability of REBT for sport performance (e.g., Wood et al., 2016), performance outcomes have not robustly been investigated and therefore the impact of REBT on performance is currently unknown. Further, the potential mechanisms for sport performance effects stemming from irrational and rational beliefs have not been suitably investigated. Third, the development of irrational beliefs in athletes should be investigated to provide a clear picture of how and when irrational beliefs emerge in athletes. This can open the door for early-years development of rational beliefs in order to avoid mental health issues stemming from irrational beliefs as the athlete progresses in their career. This article addresses each of these three areas in detail and in turn.

### The Influence of Irrational Beliefs and Rational Beliefs on Mental Health

Rational Emotive Behavior Therapy did not stem from performance literature, and like many other cognitive-behavioral approaches, REBT has been adopted by sport and exercise psychologists for use in performance settings. The origins of REBT are within clinical psychotherapeutic settings, where the chief goal is mental health. Therefore the preponderance of extant research examines mental health outcomes, and indicates that irrational beliefs lead to, and are associated with, a vast range of emotional and behavioral outcomes that undermine mental health. In this section of the current article, the author provides a review of the literature examining irrational beliefs as a risk factor for mental illness, and rational beliefs as a protective factor for mental illness. Given the dearth of research investigating irrational and rational beliefs and the mental health of athletes, the aim here is to detail the ways in which irrational and rational beliefs are associated with a broad range mental health issues that clearly could affect an athlete throughout their careers. A greater understanding of how irrational and rational beliefs contribute to mental illness is sought, with a view to proposing how future research could begin to understand this issue in sport. Although athletes have not been at the center of this research, many of the outcomes associated with and stemming from irrational beliefs could clearly hinder short-term and long-term athletic achievement, and impact upon the mental health of athletes. To present REBT as a potentially effective approach to promoting athlete mental health, it is first important to consider the wider evidence linking irrational and rational beliefs to mental health.

#### General Irrational Beliefs

A vast amount of research has been dedicated to exploring the associates of irrational beliefs in general, and the associates of the four core irrational beliefs. Bringing this large literature base together, it is possible to appreciate the expansive influence of irrational beliefs on a range of unhealthy emotional and behavioral outcomes. The theoretical structure of rational and irrational beliefs within REBT is appealing due to its symmetry and relative simplicity. But apart from their aesthetic structural appeal, rational and irrational beliefs are valuable constructs because they determine numerous cognitive, affective, and behavioral outcomes, important for mental health. Research has concentrated more on irrational beliefs than rational beliefs, perhaps reflecting a problem-focused bias, rather than a benefit-focused, in the literature concerning REBT. A review by Browne et al. (2010) highlights many unhealthy associates of irrational beliefs, such as anger, guilt and shame, and psychopathological conditions including depression, anxiety, and suicidal thoughts. Across the research that demonstrates relationships between irrational beliefs and dysfunctional emotions, the strength of the associations varies across studies (e.g., MacInnes, 2004; Bridges and Harnish, 2010a). Also, associations between increased irrational beliefs and a consequent increase in emotional or inferential dysfunction are often small (MacInnes, 2004). In MacInnes’ (2004) review of the literature he concluded that data “does not clearly indicate that there is a causal relationship”. However, it maybe that the hypothesis that irrational beliefs cause dysfunctional emotions might be true, but that the 18 studies that met the analysis criteria provide weak evidence.

In a recent meta-analysis (Visla et al., 2016), the relationships between irrational beliefs and dysfunctional emotions were examined. Eighty-three studies were included in the analyses, with a total of 16,110 participants in total across 100 different samples. The studies published between 1972 and 2014 comprised student (*N* = 34), clinical (*N* = 22), and non-clinical (*N* = 78) populations. Results revealed significant small to moderate effect sizes of general distress (*r* = 0.36), depression (*r* = 0.33), anxiety (*r* = 0.41), anger (*r* = 0.25), and guilt (*r* = 0.29). Interestingly, the association between irrational beliefs and depression was higher when a stressful event was present (*r* = 0.67, *p* < 0.001) than when not (*r* = 0.30, *p* < 0.001). Also, there was a stronger relationship between irrational beliefs and general distress when the stressful event was experimentally induced (*r* = 0.55, *p* < 0.001) as opposed to being a real stressor (*r* = 0.32, *p* < 0.001). Overall, the authors comment that the study evidenced a moderate but robust relationship between irrational beliefs and psychological distress, corroborating and extending past research (e.g., MacInnes, 2004).

Many studies show that irrational beliefs are positively associated with depression and depressive symptoms (e.g., Nelson, 1977; Ciarrochi et al., 2005), and that irrational beliefs are able to distinguish depressed from non-depressed groups (e.g., Marcotte, 1996; McDermut et al., 1997; Taghavi et al., 2006). An early study (Nelson, 1977) revealed that the strongest relationships with depression emerged for irrational beliefs that reflected a need to excel in all endeavors, that it is terrible when things are not the way one would like them to be, obsessive worry about future misfortunes, and the impossibility of overcoming the influences of past history. These four beliefs are of course salient in sport, where excellence is desirable, frequent barriers emerge that can impact success, and athletes are often judged on previous success (or failure).

The extant literature also indicates that irrational beliefs are positively related to various forms of anxiety such as trait, state, speech, social, evaluation, test anxiety in clinical and non-clinical samples (Himle et al., 1982; Deffenbacher et al., 1986), and general, phobic, and obsessive compulsives (Thyer et al., 1983). At a physiological level irrational beliefs have been associated with greater galvanic skin response (Master and Gershman, 1983), and systolic blood pressure (Harris et al., 2006), indicative of greater autonomic physiological arousal. Harris et al. (2006, p. 5) suggest that “mental rigidity” leads to autonomic rigidity reflected in systolic blood pressure increases. Examining more complex physiological parameters, Papageorgiou et al. (2006) found that irrational beliefs were positively related to C-reactive protein, interleukin-6, tumor necrosis factor-α, and white blood cell counts in healthy adults (*N* = 853), when controlling for age, sex, years of school, body mass index, physical activity status, depression levels, and dietary habits. This study suggests that irrational beliefs maybe a risk factor for cardiovascular diseases. Indeed, irrational beliefs have been shown to affect physical health, with irrational beliefs positively related to eating disorders (Möller and Bothma, 2001) such as bulimia (Phillips et al., 1997), more severe asthma symptoms (Silverglade et al., 1994), and a higher frequency of chronic illnesses (Lichtenberg et al., 1992). Indeed, irrational beliefs are also positively related to type-A coronary-prone behavior, where individuals are highly motivated and competitive, but feel near constant time pressure, and have high hostility and anger (Friedman, 1977). This has been shown to be a risk factor for cardiovascular diseases.

#### Demandingness

Demandingness in REBT is the primary irrational belief and research (see Szentagotai and Jones, 2010, for a review) indicates that demandingness is positively related to a vast array of dysfunctional emotional and behavioral outcomes. Specifically, and non-exhaustively, demandingness is associated with disordered eating (Harrington, 2005a; Pearson and Gleaves, 2006), reduced anger control (Addis and Bernard, 2002), relationship problems, social avoidance and isolation (Watson et al., 1998), decreased performance in social situations (e.g., speaking; Nicastro et al., 1999), procrastination (Beswick et al., 1988; Bridges and Roig, 1997), alcohol abuse (Hewitt and Flett, 1991), interpersonal issues (Haring et al., 2003), suicide (Blatt, 1995), and reduced task performance (Frost and Marten, 1990). It is reasonable to suggest that none of these outcomes are conducive to short or long-term athletic goal achievement.

#### Awfulizing

Awfulizing in REBT is a secondary irrational belief and has been positively related to a submissive interpersonal style (Goldberg, 1990), social isolation (Watson et al., 1998), unhealthy anger expression and suppression, and externalized behavioral disorders (Silverman and DiGiuseppe, 2001). An area highly relevant to the mental health and performance of athletes is the research surrounding pain catastrophizing (a term often used instead of awfulizing), which leads to greater pain (e.g., post-knee surgery; Pavlin et al., 2005), more distress, greater disability, poorer quality of life in response to injury, and is generally negatively related to all pain outcomes investigated such as intensity, distress, and functioning, whereas decreased catastrophizing leads to decreased pain, disability, and depressive symptoms (see Schnur et al., 2010, for a review). It is thought that the anxiety provoked by awfulizing increases perceptions of pain through inflated expectations of high pain, and can lead to negative mental states activated in anticipation and during painful experiences (Sullivan et al., 2001). Also, catastrophizing mediates the relationship between depression and pain such that higher catastrophizing promotes depressive responses to pain (Gatchel et al., 1995; Geisser and Roth, 1998). The link between irrational beliefs and injury outcomes associated with pain is highly salient to athletes, given that the vast majority (if not all) of athletes will experience injury during their careers to a greater or lesser severity and frequency.

#### Low frustration tolerance (LFT)

Low frustration tolerance is a secondary irrational belief, and research indicates that it is positively associated with aggressive expression of anger (Martin and Dahlen, 2004), reduced anger control (Moller and Van der Merwe, 1997), poor social adjustment (Watson et al., 1998), addictive behaviors (Ko et al., 2008), anxiety, depression, procrastination, and dysfunctional affect (Harrington, 2005b, 2006). The link between LFT and procrastination has received much research attention, with Ellis and Knaus (1977, p. 19) regarding LFT as “the main and the most direct cause of procrastination.” Evidence indicates that individuals with discomfort intolerance are more likely to procrastinate on boring, difficult, or demanding tasks (Milgram et al., 1988), all of which are potential demand characteristics of athletic training and competition. Individuals that find it difficult to tolerate difficulty and who experience procrastination are less likely to fulfill their achievement potential (Wilde, 2012).

#### Depreciation

Depreciation is a secondary irrational beliefs, and research has elucidated positive associations with defensiveness to negative feedback (Chamberlain and Haaga, 2001), aggressiveness (Beck, 1999), unhealthy anger expression (Martin and Dahlen, 2004), children’s depressive, fear (internalized) aggressive, and anti-social (externalized) symptoms (Silverman and DiGiuseppe, 2001), and has been associated with those who are easily threatened by criticism (Ellis and Dryden, 1997). Literature suggests that depreciation beliefs are a strong predictor of depression (Solomon et al., 2003), anxiety (David et al., 2002), and posttraumatic stress responses (Hyland et al., 2014). Depreciation reflects the notion that on the basis of one occurrence, the individual generalizes and values the self in line with the result. For example, an athlete might conclude that because they have failed in a tennis match, then they are a complete failure. In REBT it is argued that self-rating in any way is erroneous (Ellis, 1994), because there are no objective bases for arriving at global evaluations of one’s self (Sava et al., 2011). REBT is unique in that it encourages the valuation of behaviors, and the unconditional acceptance of people and the self as a whole.

#### General Rational Beliefs

Research concerning rational beliefs is less voluminous compared to research investigating irrational beliefs. Indeed, unlike research concerning irrational beliefs, the four core rational beliefs have not been examined to the same extent. This indicates a problem-orientated focus within the literature, which may reflect a more general negativity bias (Kanouse and Hanson, 1972), which refers to the tendency for things of a more negative nature (e.g., unpleasant thoughts, emotions, or social interactions; harmful/traumatic events) to have a greater effect on one’s psychological state and processes than do neutral or positive things (Lewicka et al., 1992; Baumeister et al., 2001; Rozin and Royzman, 2001). There is no reason to suppose that this tendency has not permeated the academic research concerning REBT. This issue notwithstanding, research concerning rational beliefs (see Caserta et al., 2010) indicates that higher rational beliefs are associated with greater ability to manage stress of negative life events (Ziegler and Leslie, 2003; David et al., 2005b), anxiety (Himle et al., 1982), job stress (Tan, 2004), bereavement (Boelen et al., 2004), and adjustment (Lee et al., 2004; Ireland et al., 2005). It has also been found that the use of rational self-statements during pressured tasks leads to less self-reported anxiety (Rosin and Nelson, 1983) and emotional distress (Cramer and Kupshik, 1993) than irrational self-statements, which may have implications for athletes in terms of how they structure and frame their self-talk.

#### Unconditional Self Acceptance (USA)

Unconditional self acceptance is a specific rational belief that reflects unconditional regard for oneself despite undesirable behaviors and adverse events (e.g., rejection, failure). So where irrational self-depreciation beliefs involve assigning low self-value and self-worth on the basis of an event, USA involves accepting oneself regardless of the adversity (Hill et al., 2008). Davies (2008a) found that USA was most strongly negatively related to self-depreciation, need for achievement, and need for approval. Further, participants primed with irrational self-statements showed reduced USA, and those primed with rational self-statements showed increased USA (Davies, 2008b). These studies show that USA is a rational belief that contrasts with irrational beliefs. USA is negatively related to depression and anxiety (Chamberlain and Haaga, 2001; Scott, 2007), and low USA can lead to self-blame and self-criticism (cf. Hill et al., 2008) and may increase the propensity for narcissism, downward social comparison (a person looks to another individual or group that they consider to be worse off than themselves in order to feel better), and self-centeredness (Neff, 2003).

Research also shows that USA is negatively related to self-oriented, other-oriented, and socially prescribed perfectionism (Flett et al., 2003). Further, Flett et al. (2003) found that USA mediated the association between socially prescribed perfectionism and depression, and other-oriented perfectionism only affected depression through its association with low USA. This is important because perfectionism has been found to lead to a range of dysfunctional emotions (Flett et al., 2003), and similarly in athletes has been associated with psychological distress, motivational deficits, negative thoughts, anxiety, and low confidence (see Flett and Hewitt, 2005, for a review). Furthermore, Flett et al. (2003) found strong links between self-oriented perfectionism and self-worth beliefs. The authors note that adolescents with high levels of self-oriented perfectionism acknowledged that their self-worth is based on how they are evaluated by themselves and by other people and whether approval and recognition are forthcoming from others. This is salient in sport where self and other evaluation is part and parcel of the context within which athletes are performing. Indeed, perfectionists often have a contingent sense of self-worth (e.g., DiBartolo et al., 2004) often equating making mistakes with their sense of self-worth (Bernard et al., 2006). It is difficult to imagine any athlete able to navigate the complex performance environment without making mistakes or facing set backs that may impinge on their sense of self-worth.

#### Critical Summary

The extant research shows that irrational and rational beliefs are related to a vast array of outcomes relevant to mental health. Although the research has not considered athletes, clearly the mental health outcomes can affect athletes within and outside of their sport participation. Only one study has directly examined the relationship between irrational beliefs and mental health outcomes, which revealed that irrational beliefs predicted increases in physical and emotional exhaustion (a dimension of burnout) in Gaelic football athletes over an 8-week period (Turner and Moore, 2015). This finding is inline with past research outside of sport linking irrational beliefs to greater burnout (e.g., Balevre et al., 2012). But clearly more longitudinal, large-scale, studies are required in order to investigate fully the relationships between irrational beliefs and dysfunctional responses, especially focusing on athletes. The lack of research assessing outcomes associated with rational beliefs needs to be addressed for any meaningful conclusions to be draw as to the benefits of rational beliefs, as opposed to the benefits of low irrational beliefs. This is an important distinction because irrational and rational beliefs are relatively orthogonal, and low irrational beliefs do not necessarily mean high rational beliefs (i.e., they do not correlate highly; Ellis et al., 2010). Therefore a relatively separate and equitable research literature is required for both irrational beliefs and rational beliefs, which is currently not present.

Although there is a positive association between irrational beliefs and clinical and non-clinical mental health outcomes (e.g., McLennan, 1987; Prud’homme and Barron, 1992; Macavei, 2005), past research has been hindered by the way in which irrational beliefs have been assessed. There are various psychometric measures of irrational beliefs, many of which have fundamental limitations (see Bridges and Harnish, 2010b, for a review) thus casting doubts over the findings from previous research. In response to a critical review of irrational beliefs assessments (Terjesen et al., 2009) the development of new measures has strengthened the assessment of irrational beliefs, with one inventory specifically developed for use in performance settings such as sport (irrational performance beliefs inventory, iPBI; Turner et al., 2016). The iPBI has also shown that higher irrational beliefs are positively related to trait depression, trait anger, and trait anxiety.

### The Influence of Irrational Beliefs and REBT on Athletic Performance

As evidenced in the extant research, it is unfavorable to hold irrational beliefs due to the associated maladaptive emotional and behavioral consequences that impede mental health. So it is tempting and indeed reasonable to assume that within a performance setting such as sport, irrational beliefs would be deleterious for goal attainment. However, there is little evidence that irrational beliefs interfere with athletic performance or that rational beliefs facilitate athletic performance (e.g., Turner, 2014). In addition, there is little evidence that irrational beliefs lead to the same emotional and behavioral outcomes in athletes as they do in non-athletes. It is sensible to hypothesize that irrational beliefs are as much of an issue in athletes as they are in non-athletes, as irrational beliefs are evident in almost all societal and cultural domains (Ellis, 1976), but sport research to date is yet to determine this.

The research conducted in sport does show that REBT is an effective intervention for reducing irrational beliefs and performance anxiety (Elko and Ostrow, 1991; Yamauchi and Murakoshi, 2001; Larner et al., 2007; Turner and Barker, 2013), and one case-study demonstrates the improved competitive performance of an archery athlete (Wood et al., 2016). This research employs applied research methods offering support for the use of REBT with athletes via a traditional one-to-one counseling approach. However, little research has included objective performance markers to assess the effects of REBT on athletic attainment in the short-term or long-term. That is, it is not known whether REBT enhances skill execution or kinematic aspects of sport performance, or aspects that allow long-term goals to be attained (e.g., motivation, emotion regulation). Anecdotally practitioners have reported observing gains in the performance of the athletes they have applied REBT with (Bernard, 1985; Marlow, 2009; Turner and Barker, 2014; Turner et al., 2014), but this does not constitute objective or even empirical evidence for the performance facilitating effects of REBT or rational beliefs.

Because of the dearth of REBT research in sport, it is necessary to look to research that assesses irrational beliefs, REBT, and performance in other domains. In the meta-analysis by Gonzalez et al. (2004), REBT was shown to have a moderate positive effect on adolescent academic (Grade Point Average) performance (mean *Z*^r^ score of 0.49). The research included in the meta-analysis that used GPA as a performance marker have the advantage of including an objective indication of performance. However, it is not possible to pinpoint what it was about the REBT delivered that increased performance. Some research suggests that irrational beliefs are inversely related to reading ability, with speculation that rational beliefs may be related to verbal intelligence (Prola, 1988). It could also be argued that positive changes in behaviors, such class cutting (Block, 1978) and increased effort (Kachman and Mazer, 1990), could contribute to GPA, but there are a myriad of extraneous factors that contribute to assessment performance for school students. Therefore it is difficult from the above research to extract translatable findings to sport, as the mechanisms between reductions in irrational beliefs and performance were not investigated.

Research that more directly assess performance in relation to irrational and rational beliefs adopts laboratory experimental approaches. For example, participants on approach to a mirror-tracing task given irrational self-talk statements (e.g., “If I don’t perform perfectly well next time it will prove I’m stupid,”) recorded the most errors in the task, and performed slower than the rational self-talk group (e.g., “Mistakes don’t mean I’m stupid”; Schill et al., 1978, p. 4). The irrational self-talk group evidenced the worst performance efficiency in the task. In a similar study of mirror tracing performance (Bonadies and Bass, 1984) it was found that participants given rational self-talk statements were significantly more accurate than those given irrational self-talk statements, and a control group. The irrational self-talk group and control group were as inaccurate as each other, suggesting that rational self-talk is beneficial for performance, rather than irrational self-talk being damaging for performance. From these laboratory studies, it could be suggested that movement efficiency in laboratory-based tasks can be harmed by adopting irrational self-talk. However, self-talk is considered to be effective for cognitive control but is not necessarily reflective of deeply held beliefs (Bunker et al., 1993).

With the meta-analysis (Gonzalez et al., 2004) and the laboratory studies (Schill et al., 1978; Bonadies and Bass, 1984) considered, there is little research evidence that irrational beliefs could harm athletic performance. The notion that REBT can help athletic performance comes from anecdotal and case-study sources, supported by REBT theory and research purporting that rational beliefs lead to functional and adaptive emotions and behaviors that facilitate goal attainment. Viewed through a humanistic and hedonic lens, it could be argued that athletes who feel better will perform better. For example, mood states are able to predict athletic performance, whereby athletes with lower depression, anger, and tension are more likely to perform well (e.g., Beedie et al., 2000). In addition, recent work in the field of REBT (Dryden, 2007; Neenan, 2009) and resilience (Fletcher and Sarkar, 2013) intimates that there exists some symmetry between REBT and the concept of resilience that may help to better understand and develop resilience.

Resilience can be considered to be a process of adapting well in the face of adversity, recognizing that emotional distress is very much a part of becoming resilient (see Sarkar et al., 2015), and that coming back from adversity is not necessarily an immediate occurrence (American Psychological Association, 2004; Dryden, 2007). There is a need in sport for techniques that promote resilience, particularly those that include the minimization of catastrophic thinking, the challenging of counterproductive beliefs, and the encouragement of cognitive restructuring (Fletcher and Sarkar, 2012). The minimization of catastrophic thinking and the challenging of counterproductive beliefs are both central to the aims and process of REBT, with cognitive restructuring particularly important for the endorsement of rational beliefs over irrational beliefs. Indeed, the capacity for rational beliefs to promote resilience has been met with the formulation of an Athlete Rational Resilience Credo (Turner, in press), which encourages successful adaptation to adversity in athletes, and based on the work of Dryden (2007 p. 219), presents “a set of beliefs, which expresses a particular opinion and influences the way you live.” The links between irrational and rational beliefs and resilience, and the use of REBT to promote resilience, are yet to be fully explored but offer potentially fruitful areas of future research that could help to elucidate the links between irrational and rational beliefs, and sport performance.

#### The Potential Facilitating Effects of Irrational Beliefs

Despite the notion that irrational beliefs are harmful for performance, many athletes and coaches the author works with are adamant that irrational beliefs can help athletic performance, such that they are reluctant to shed their irrational beliefs for rational beliefs. To be candid, there is no hard evidence suggesting that irrational beliefs harm athletic performance, and of course there is insufficient evidence supporting the opposite notion that irrational beliefs help athletic performance. In the Schill et al. (1978) study, some individuals allocated to the irrational self-talk group did perform well, in contrast to the overall findings, a finding that should not be ignored. Indeed, there may be a self-reinforcing thrill achieved when people think irrationally, and distortion and exaggeration can be exciting and may elicit the attention or sympathy of others (Digiuseppe et al., 2014). Thus it may be that people experience short-term advantages by thinking irrationally. Once one thinks oneself to be worthless, one is legitimized to not put effort into achieving ones goals (Backx, 2012). In the absence of more evidence relating irrational beliefs to performance, it is perhaps understandable why coaches and athletes are vigilant when it comes to challenging their own and their athletes’ beliefs. While the evidence overwhelmingly suggests that irrational beliefs are deleterious to mental health, it cannot be discounted that irrational beliefs may be acutely helpful for motor performance. Perhaps a ‘must win’ belief provides an extra impetus to exert more effort on a given task, or provides a focus on what is to be done to get the edge over an opponent within a competition, for example. Indeed, some athletes adopt negative self-talk as a useful strategy for performance as it may contain motivating properties (Goodhart, 1986). It might be helpful to draw on motivation theory to understand this apparent paradox, such as the self-determination theory (Deci and Ryan, 1985, 1991; Ryan and Deci, 2000, 2002).

In self-determination theory individuals are considered to be intentional organisms motivated to achieve differing objectives. Hence, Deci and Ryan (1985) posited three types of motivation that capture the different reasons for individual engagement in achievement situations; intrinsic motivation, extrinsic motivation, and amotivation. Intrinsic motivation, various extrinsic motivations (external regulation, introjected regulation, and identified regulation), and amotivation reflect a self-determination continuum. Excellent papers can be read on the intricacies of the self-determination continuum (e.g., Standage et al., 2005), however, for the purposes of the current article, it is sufficient to understand that intrinsic motivation refers to engagement in an activity for its own sake (pleasure, interest, and satisfaction from activity itself), and as an individual’s motives approach extrinsic motivation on the continuum, the more that engagement in the activity is directed by separable outcomes such as rewards or punishments.

Of particular interest due to its potential relationships with irrational beliefs, is introjected regulation, which reflects moderately low self-regulation. Introjected regulation is characterized by an individual internalizing external regulations (Ryan and Deci, 2002), where the direction for action is controlled by self-imposed sanctions such as to avoid shame and guilt, or attain ego enhancement such as pride. That is, introjected regulation represents regulation by contingent self-esteem (Deci and Ryan, 1995, 2000). Standage et al. (2005 p. 413) give an example that resonates with REBT, stating that “an example of introjected regulation would be a student that participates in an after school physical activity program, not because she/he wants to, but because the student feels that she/he should, because that is what ‘good students’ do.” This reflects both demandingness beliefs (“should”) and global evaluations (“good students”). Indeed, the perception that one should or ought to engage in an activity is considered a hallmark of introjected regulation (e.g., Gillison et al., 2009).

The potential relationship between irrational beliefs and introjected regulation is important because it may begin to elucidate how irrational beliefs could have both positive and negative effects on athletic performance. Introjected regulation is an external regulation, and as people internalize regulations and assimilate them to the self, they experience greater autonomy in action (Deci and Ryan, 2000). More autonomous extrinsic motivation has been associated with more engagement (Connell and Wellborn, 1991), better performance (Miserandino, 1996), lower dropout (Vallerand and Bissonnette, 1992), higher quality learning (Grolnick and Ryan, 1987), and better teacher ratings (Hayamizu, 1997), among other outcomes (see Deci and Ryan, 2000, for a review). In one study conducted with students (Ryan and Connell, 1989), higher external regulation was associated with less interest, less value, and less effort toward achievement, and higher tendencies to disown responsibility for negative outcomes. Specifically, introjected regulation was positively related to expending more effort, but it was also related to feeling more anxiety and coping more poorly with failures. Therefore, irrational beliefs reflecting introjected regulation may inspire effort due to internal pressure (“I have to”), but may have implications that undermine performance in the longer-term.

Echoing the above, Harrington (2005b) suggests that in some circumstances irrational beliefs about high standards may increase functional behavior, however, he also recognizes that this may lead to psychological problems in other areas due to increased workload and stress. So although irrational beliefs may inspire effort, there are numerous risk factors that could emerge as a result. Are we as practitioners comfortable with the idea that athletes and coaches may promote irrational beliefs for short-term performance, when we know that long-term mental health may be compromised? There is enough evidence that links irrational beliefs to harmful emotional and behavioral consequences to drive those involved in sport to endeavor to reduce irrational beliefs in athletes, and find alternate methods of motivating optimal performance.

#### Double-Think

Perhaps irrational performance beliefs are more complex than being either rational or irrational. Some athletes the author has worked with are capable of using irrational thoughts for acute performance situations, while harboring robust rational beliefs as part of their philosophy of success and failure. Self-talk in the moment does not necessarily reflect core beliefs. In other words, it is possible for athletes to partake in what George Orwell called “double think” in his book 1984, which is described as “…the power of holding two contradictory beliefs in one’s mind simultaneously, and accepting both of them.” (Orwell, 1949, p. 32). Orwell goes on to describe doublethink in the following way:

“To tell deliberate lies while genuinely believing in them, to forget any fact that has become inconvenient, and then when it becomes necessary again, to draw it back from oblivion for just so long as it is needed….” (Orwell, 1949, p. 32).

Holding contradictory ideas, such as irrational and rational beliefs, is also akin to the concept of cognitive dissonance (Festinger, 1957), which may result in discomfort due to simultaneous endorsement of two contradicting beliefs. Supposedly, people are motivated to reduce this dissonance, and therefore holding both irrational and rational beliefs about the same situation may be difficult (Festinger, 1957). For example, a marathon runner in the final mile may use the self-talk “I want to get my personal best and therefore I have to, and it would be awful if I did not” which may inspire a final burst of enthusiasm toward the home straight. However, the runner may harbor a core belief that recognizes that “I want to get my personal best, but that does not mean I have to, and it would be bad but not awful if I did not” that directly opposes the self-talk, but this core belief is not useful or salient in that context. When there is dissonance such as the above example an individual can conveniently ignore the contradiction because in the moment the irrational self-talk is achieving the desired results of increased performance. For example, a smoker knows that smoking is deadly, but can justify smoking because it feels good and helps them to relax in the moment. So to reduce the dissonance between irrational and rational beliefs, athletes can view irrational self-talk as helpful in the moment, thus reducing the conflict between the contradictions. Novak Djokovic, professional tennis player with 12 Grand Slam singles titles (at the time of writing), gives a nice example of this, where he says that “Tennis is my life, obviously; I need to focus, I need to win. But it’s not the only thing. I’m not going to play forever” (Wilbon, 2012). Here, Djokovic expresses the rigid and extreme need to succeed, but also expresses a flexible and non-extreme belief that tennis is not the only thing. This ability to use irrational self-talk while holding rational core beliefs relies on the athletes meta-cognitive ability to introspect on their thought processes (Metcalfe and Shimamura, 1994), and be able to understand that different beliefs are appropriate for different circumstances. Athletes unable to relinquish their irrational self-talk when they step off the court may find it difficult to endorse rational beliefs, and therefore may be at risk from the many mental health outcomes highlighted in this article.

#### Critical Summary

The question of whether irrational beliefs influence athletic performance or not, is a complex but valuable one, for which little research exists. There are reasons to believe that irrational beliefs could actually help performance particularly in the short-term if athletes are able to recognize the differences between their self-talk and their core beliefs, but that long-term sport performance is unlikely to benefit from holding irrational beliefs in part due to the deleterious implications on mental health. If irrational beliefs are in some way helpful in the short-term, or can facilitate acute skilled performance, we must ponder the potential costs of such beliefs long-term. Enough evidence exists regarding mental health that should dissuade the promotion of irrational beliefs. Thus even if irrational beliefs can in some situations help performance, it is questionable whether irrational beliefs should ever be encouraged. These conjectures are based on anecdotal evidence, theory and inferences made from, at times, disparate research, and it has to be stressed that little data currently exists that could empirically support, or refute, these conjectures. It is hoped that the ideas above promote further investigation by researchers and practitioners.

### The Origins and Development of Irrational and Rational Beliefs in Athletes

The current review has presented evidence that attests to strong links between irrational beliefs and poorer mental health. It is argued that the links between irrational and rational beliefs and mental health outcomes exist because of the associated functional (healthy) and dysfunctional (unhealthy) emotional and behavioral consequences that are driven by the beliefs. Because irrational beliefs lead to a range of UNEs that undermine mental health, it is important to understand how irrational beliefs are developed and propagated, so that interventions and strategies can be developed to dissuade the development of irrational beliefs, and promote rational beliefs, before formal REBT is required.

#### Nature vs. Nurture

Like many psychological constructs, there is a debate as to whether irrational beliefs are driven by nature (we are born irrational) or by nurture (we learn irrationality). Ellis (1976) intimated, and Ruth (1992) propagated, that there is a biological basis or innate tendency for rational and irrational beliefs, such that it is in our nature to develop both rational and irrational beliefs. Ellis wrote, “humans have a strong tendency to needlessly and severely disturb themselves, and that, to make matters much worse, they also are powerfully predisposed to unconsciously and habitually prolong their mental dysfunctioning and to fight like hell against giving it up” (Ellis, 1987, p. 365). Ellis (1987) suggests that humans are predisposed to develop such beliefs, thus explaining why they are difficult to change, and why they persist despite cultural teachings and self-awareness. Ellis’ core argument was that all humans at some point believe, think, and act in ways that are self-destructive, reflecting the illogicality of holding such beliefs, thoughts, and behaviors (Sharf, 1996). This is evident across all known social and cultural groups. He argues that all societal and cultural institutions promote demandingness, awfulizing, low-frustration tolerance, and depreciation to greater or lesser extents.

Ellis recognizes that societal and cultural irrationalities are driven by the teachings of significant others, and makes the point that we ourselves have a propensity to willingly receive and internalize these irrationalities and continue to perpetuate them for the rest of our lives. Irrational beliefs are exacerbated by those around us whom we look to for guidance on how to live our lives (Sharf, 1996), but irrational beliefs are so ubiquitous that they cannot possibly be entirely learned. Indeed, even when society dissuades certain self-destructive behaviors, humans are resistant to rationality and constantly think and act in ways that are contrary to self-propagation and the development of the human race. We give up many erroneous beliefs as we develop and mature, but irrational beliefs maintain.

In sum, Ellis and others suggest that we are predisposed to irrational beliefs, but that society has a part to play in teaching us these beliefs. Depending on these biological and social factors, individuals can vary in their vulnerability to psychological disturbance (Sharf, 1996). Ultimately, society drives irrationality, and we as humans develop irrational beliefs as a function of our biological tendency to do so. So assuming that athletes are normal human beings (which of course they are), we have to look to the culture of sport to start to explain sport-related irrational beliefs in athletes (the irrational beliefs of the vast number of individuals involved in sport who are not athletes will be ignored for the purposes of this paper). It is difficult to understand or estimate the prevalence of irrational beliefs in athletes due to the dearth in relevant literature. Typically, prevalence research randomly selects a sample from the population in question (e.g., athletes) and provides an estimation of the problem for the population (Boyle, 1998). This approach has not been taken so far within the REBT literature concerning sport. As previously reviewed, most of the extant literature is applied in nature and is more concerned with measuring the effects of REBT on small numbers of athletes. However, there is a need to understand the extent to which athletes hold irrational and rational beliefs, and whether this differs from more prominently studied populations (e.g., students, clinical samples). At present, it is not known whether athletes are more or less likely to endorse irrational and rational beliefs compared to non-athlete populations. Therefore, it is very difficult to grasp the extent of the problem.

#### The Language of Sport

Although data do not exist that reveal the prevalence of irrational and rational beliefs in athletes, when sport is viewed through the lens of the media, irrational beliefs are evident in the language used in the daily sporting narrative. The way that sport is portrayed in the media would have us believe that the very nature of sport is irrational, with barely a week going by without irrational language being used to inappropriately describe the on and off field exploits of athletes and coaches. To use a recent example, Louis van Gaal (Manchester United Football Club manger) was asked prior to the 2015 Boxing Day match against Stoke City Football Club if it is a “must-win” and van Gaal said “Yes, I think so because when you have lost three times in a row to you need to win.” He said “We have to focus on that match and we have to win that match.” Manchester United lost that match 2–0, which apparently was a “must-win” (Sheen, 2015). In contrast, Jurgen Klopp often uses more rational language, and after failing to win in his first three matches in charge of Liverpool Football Club, he said:

“This is not the end of the world. We conceded a goal near the end, and it felt like the end of the world, but it is not the end of the world…I hope I’m not the only person in the stadium who thought: ‘This is not the end of the world.’ We can work on this…Of course, it is not the best moment for us, because we wasted a lot of energy. Southampton haven’t lost away from home, so we had to work hard…You score the goal and you want to win, but it didn’t happen for us today. Football is not a fairytale. Sometimes we can write stories like this but it doesn’t always happen” (Agence France-Presse, 2015).

Therefore, if the prevalence of irrational and rational beliefs can in some part be reflected in the utterances of athletes and coaches, then a deeper analysis of this phenomenon is warranted. Of course, what athletes and coaches say to the media is not necessarily indicative of deeply held core beliefs, and caution should always be paid to the interpretation of media sound bites. But it is recognized that common cultural stereotypes in our language, our stories, our songs, play a part in developing rational and irrational beliefs (Digiuseppe et al., 2014). It could be argued that sport is especially endorsing of irrationality, where irrationality is openly propagated and often boasted about. Indeed, the irrational notion of “terrible performances” and “must win games” is common parlance in many sports, and even used as a badge of honor to demonstrate how much an individual cares about their performance. The language used to report sport may play a role in not only reflecting irrational beliefs, but may also play a role in developing irrational beliefs.

Athletes, just like any other human being, populate a complex social world. To navigate this social world of coaches, teammates, support staff, opponents, supporters (and non-supporters), the media, and of course friends and family, the reciprocal communication of information is important. An athlete takes instruction, advice, guidance, opinions, and criticism on a daily basis, but also may deliver similar information to others on a typical day. This is important because it suggests that people are influenced by language used in communication with others and oneself, and therefore when imprecise language is used (such as the verbal expression of rigid, extreme, and illogical beliefs) this can augment imprecise thinking (Dryden, 2015). This is in line with General Semantics Theory (Korzybski, 1933), and suggests that the language used to communicate with athletes is a powerful source of information guiding thought processes and subsequent emotional and behavioral responding. Therefore, even if key stakeholders of an athlete’s mental health do not really believe the irrational beliefs they portray in their use of language, the communication of such information contributes to the beliefs of athletes.

The examination of language within sport settings may be a fruitful area for future research, partly because it offers the advantages of representing rigid irrational beliefs in the symbolic form of language where the belief is more easily examined and manipulated (Dryden, 2015) compared to deeper level beliefs (e.g., schemas; David et al., 2002). If irrational beliefs are developed through cultural learning, key stakeholders in the development of team and organizational cultures that surround athletes should be investigated. This investigation should focus on the language used by coaches, managers, support staff, parents, and athletes, in training and performance settings, and whether this language contains content symbolic of irrational beliefs. Also, shifts in athlete beliefs during and after the manipulation of this language should be explored to understand the impact of irrational and rational language on beliefs. That is, controlled experimental and field studies should be undertaken that directly manipulate rational and irrational language to understand the extent to which this language triggers the development of rational and irrational beliefs, and associated emotional and behavioral maladaptation.

#### Critical Summary

If it is apparent through empirical research that athlete irrational beliefs are driven by socialization, then it is possible to start to combat irrational beliefs in athletes by equipping key stakeholders with information and education concerning the dissuasion of irrational language, and the promotion of rational language. This may involve systematically engaging with coaches, parents, support staff, and performance directors, to help challenge the cascade of irrational language that athletes are exposed to all too frequently. Of course, the most direct way to challenge irrational beliefs in athletes to work directly with those athletes. However, cultural changes within organizations can have a more long-lasting and broader effect (Katzenbach et al., 2012) on a larger number of athletes that may go some way to reducing the need for individual REBT with athletes. If we are to meaningfully reduce the development of irrational beliefs in athletes, we must first understand how they emerge and what factors drive their development. Then targeted interventions can be formulated and applied within sporting organizations with athletes and key stakeholders in athlete mental health.

## Key Questions for Future REBT Research in Sport

In this section, the themes highlighted throughout this article are brought forward and key questions for future research are proposed. These questions should guide the future investigation of REBT in sport to enable stronger conclusions to be drawn about the value of REBT theory and practice as applied to the mental health and performance of athletes. The key questions reflect what is currently known about irrational beliefs and REBT from research outside of sport, and bring forth the most salient areas for future research in sport.

### Do Irrational Beliefs and Rational Beliefs Influence the Mental Health of Athletes?

The extant research demonstrates that, in non-clinical and clinical populations, irrational beliefs are positively related to mental illness (and symptoms of mental ill health), whereas rational beliefs are positively related to mental health. However, it is not yet known whether these well-supported assertions hold true in sport settings, in athletes. This is surely the chief research question to emerge from this article and should be of paramount concern to researchers investigating REBT in sport. Researchers should also begin to investigate whether the four core irrational beliefs separately predict sporting emotions and behaviors in training and competitive contexts. For example, an understanding of irrational beliefs, and particularly awfulizing (catastrophizing) in athletes may help to manage pain more effectively through rehabilitation programs. In particular, longitudinal research is needed to understand to what extent irrational and rational beliefs predict changes in mental health outcomes in the long-term. There is a huge array of mental health outcomes that could be assessed, and research should focus on objective markers such as physiological reactivity and behavioral indicators (e.g., injury, physical illness). Also, moving beyond correlational research, future studies should seek to distinguish between athletes with lesser and greater mental health, and apply REBT to specifically address mental health outcomes.

### Do Irrational Beliefs and Rational Beliefs Influence Athletic Performance?

Current knowledge about the relationship between irrational and rational beliefs and athletic performance stems from disparate research, anecdotal reflections, and self-reported athlete perceptions. The current review debates a potentially complex picture of how irrational beliefs may relate to performance that has the potential to generate many future research studies. For example, can irrational beliefs be beneficial for performance in the short-term compared to the long-term? Also, can athletes engage in cognitive dissonance that allows them to utilize irrational beliefs for acute performance purposes, but endorsing rational beliefs as a more general life philosophy? Further, the motivational properties of irrational and rational beliefs for sport performance need to be better understood. The present review offers some theoretically plausible ideas that marry self-determination theory constructs with irrational beliefs, but research should examine these using meditational and longitudinal methods to understand how irrational beliefs predict changes in motivation regulation. Also, the notion of resilience in sport is becoming an important construct in research, and REBT addresses some of the main recommendations for resilience training. Therefore, research exploring the use of REBT to enhance the resilience of athletes should be undertaken, with a view to understanding the contribution of REBT to long-term athletic achievement.

### How Are Irrational Beliefs Developed in Athletes?

This article suggests that the development of irrational beliefs in athletes is likely to be driven by athlete socialization into competitive environments, while as human beings athletes have an innate tendency to develop irrational and rational beliefs. In fact, it is argued in this review that sport is especially promoting of irrational beliefs especially through language, and therefore, the culture of sport, both from an individual sports organization and a broad athletic community perspective, should be investigated. Therefore primarily researchers should conduct investigations that first seek to confirm the nature of irrational beliefs (e.g., are irrational and rational beliefs schemas?), and then seek to understand how irrational beliefs are developed. This research needs to occur in the general population as well as within sport samples, as there may be differences in how irrational and rational beliefs are perpetuated in sport compared to other endeavors (performance and non-performance). Understanding how irrational beliefs are developed in athletes will guide the psychosocial development of athletes, and inform the application of REBT in sport to address mental health outcomes throughout an athlete’s career. This important question may be aided by researchers and practitioners with expertise in developmental psychology, and the more general formation of beliefs, and therefore would no doubt be a fruitful area for multidisciplinary research.

## Conclusion

The purpose of this article was to present three keys areas that would allow further understanding of irrational beliefs and REBT within sport with a particular focus on mental health. Due to REBT’s focus on mental health, this article examined the evidence linking irrational and rational beliefs with mental health outcomes. As part of this examination, athletic performance, emotional responding, and the development of irrational beliefs, were considered. REBT is proposed as an important framework for use with athletes. Through understanding the links between irrational and rational beliefs, and mental health, this article offered a number of research questions that should be addressed by researchers. In addition, the application of REBT should be promoted to neophyte practitioners, and the applied work of practitioners using REBT should be more frequently reported in primary research and case-study outlets, so that a shared understanding of how REBT can be applied in sport is garnered. Overall, it is hoped that this article fuels the interests of researchers, students, and practitioners, so that the value of REBT for the promotion of mental heath in sport is recognized and endorsed more widely.

## Author Contributions

The author confirms being the sole contributor of this work and approved it for publication.

## Conflict of Interest Statement

The author declares that the research was conducted in the absence of any commercial or financial relationships that could be construed as a potential conflict of interest.
